# An integrated multi-omics analysis of topoisomerase family in pan-cancer: Friend or foe?

**DOI:** 10.1371/journal.pone.0274546

**Published:** 2022-10-26

**Authors:** Xin Zhou, Guixiang Yao, Jin Zhang, Jiasheng Bian, Guanghao Li, Jianfeng Xu

**Affiliations:** 1 Department of Biopharmaceutics, College of Food Science and Technology, Shanghai Ocean University, Shanghai, China; 2 Department of Cardiology, Qilu Hospital, Cheeloo College of Medicine, Shandong University, Jinan, Shandong, China; 3 Department of Bone and Soft Tissue Tumors, Tianjin Medical University Cancer Institute and Hospital, Tianjin, China; 4 Department of Urology, Shandong Cancer Hospital and Institute, Shandong First Medical University and Shandong Academy of Medical Sciences, Jinan, Shandong, China; The University of Texas MD Anderson Cancer Center, UNITED STATES

## Abstract

**Background:**

Topoisomerases are nuclear enzymes that get to the bottom of topological troubles related with DNA all through a range of genetic procedures. More and more studies have shown that topoisomerase-mediated DNA cleavage plays crucial roles in tumor cell death and carcinogenesis. There is however still a lack of comprehensive multi-omics studies related to topoisomerase family genes from a pan-cancer perspective.

**Methods:**

In this study, a multiomics pan-cancer analysis of topoisomerase family genes was conducted by integrating over 10,000 multi-dimensional cancer genomic data across 33 cancer types from The Cancer Genome Atlas (TCGA), 481 small molecule drug response data from cancer therapeutics response portal (CTRP) as well as normal tissue data from Genotype-Tissue Expression (GTEx). Finally, overall activity-level analyses of topoisomerase in pan-cancers were performed by gene set variation analysis (GSVA), together with differential expression, clinical relevancy, immune cell infiltration and regulation of cancer-related pathways.

**Results:**

Dysregulated gene expression of topoisomerase family were related to genomic changes and abnormal epigenetic modifications. The expression levels of topoisomerase family genes could significantly impact cancer progression, intratumoral heterogeneity, alterations in the immunological condition and regulation of the cancer marker-related pathways, which in turn caused the differences in potential drugs sensitivity and the distinct prognosis of patients.

**Conclusion:**

It was anticipated that topoisomerase family genes would become novel prognostic biomarkers for cancer patients and provide new insights for the diagnosis and treatment of tumors.

## Introduction

Multi-omics dysregulations abnormalities are a major cause of cancer, such as genetic alterations, differential DNA methylations, and transcriptomic and metabolic disorders [1]. With the increasing numbers of cancer patients worldwide, cancer has become a major public health problem threatening human health. In accordance with the Global Cancer Statistics (GLOBOCAN) for 2020, it is estimated that the new cancer cases will be 19.3 million and cancer-related deaths will be nearly 10 million [2,3]. Over the past few decades, cancer prevention, screening, diagnosis, and comprehensive treatment have met with tremendous success in various tumor types. It is necessary, however, to refine studies concerning the clinical outcome of most cancers [4]. The proliferation of high-throughput sequencing and advances in bioinformatic techniques made it possible to systematically study roles of hub tumor-related genes in tumorigenesis and progression [5]. It is noteworthy, however, that the altered genes vary considerably across tumor types and samples of the same tumor [6]. It is therefore essential to understand the omics-level alterations in different cancer types in order to identify novel therapeutic targets and vulnerabilities.

An essential nuclear enzyme, DNA topoisomerase regulates and controls the topological state of DNA during transcription. It comprises two subfamilies: type I topoisomerase (TOP1) induces single-stranded breaks, whereas type II topoisomerase (TOP2) induces double strand breaks. According to differences in amino acid sequence structures and/or phylogenetic relationships, these topoisomerase can further divide into type IA, IB, IC, IIA and IIB [7]. Human cells encode six different types of DNA topoisomerases: IA enzymes (TOP3α andTOP3β), IB enzymes (TOP1 and TOP1mt) and IIAenzymes (TOP2α and TOP2β) [8]. Cumulating evidence suggests that DNA topoisomerases play an important role in cancer initiation and progression due to their essential roles in triggering, controlling, and modifying a wealth of topological DNA problems during cell proliferation, differentiation [9–13]. Therefore, it is of importance to understand the expression patterns, potential molecular mechanisms, functional roles and prognostic impact of topoisomerase family genes in tumorigenesis and progression. So far, however, to our knowledge, previous studies about DNA topoisomerase were limited to certain cancer types while few studies have addressed in-depth the comprehensive molecular characterization of topoisomerase family genes from a pan-cancer perspective.

In this study, we comprehensively and systematically performed an in-depth exploration of topoisomerase family genes by using pan-cancer multi-omics data from TCGA. In the study, we discovered that gene alterations and epigenetic modifications in topoisomerase family genes could be associated with abnormal expression of these genes and affect prognoses of cancer patients. In addition, expression levels of topoisomerase family genes were significantly correlated with molecular subtypes, clinicopathologic stage, immune subtypes, regulation of cancer-related pathways, sensitivity of anticancer drugs and prognostic outcomes in various types of cancer. In other words, topoisomerase family genes may be potential therapeutic targets of multiple cancers and provide new clues for the scientific research.

## Materials and methods

### Data sources

Topoisomerase family gene list was downloaded from MSigDB gene set “GOMF_DNA_TOPOISOMERASE_ACTIVITY”(http://www.gsea-msigdb.org/). To investigate the gene expression of topoisomerase family genes in different normal tissues obtained from healthy individuals, we downloaded RNAseq data (data release version 7.0) normalized by TPM (Transcripts per Million) from GTEx portal (https://commonfund.nih.gov/GTEx/). The GTEx expression dataset, consisting of 11,688 samples, contains the expression profiles of 56,202 genes from 30 organs (53 tissues) that were donated by 714 healthy individuals. Multiomics pan-cancer datasets were obtained from TCGA database (https://portal.gdc.cancer.gov/), including mRNA seq level 3 data (n = 10,995), clinical data (n = 11,160), Illumina HumanMethylation 450k level 3 data (n = 10,129), single nucleotide variation (SNV) data (n = 10234), copy number variation (CNV) data (n = 11,495) and miRNA transcript expression data(n = 9105). Reverse-phase protein array (RPPA) data (n = 7876) were obtained from the cancer proteome atlas (TCPA) database (https://tcpaportal.org/tcpa/index.html). TCPA RPPA data are all from TCGA samples across 32 cancer types. IC50 drug data of 481 small molecules in 1001 cell lines from the CTRP (https://portals.broadinstitute.org/ctrp/) database were collected to investigate the correlation between gene expression of topoisomerase family genes and drug sensitivity.

33 TCGA cancer types and 30 GTEx normal tissues were included in the study. Cancer type: Acute myeloid leukemia (LAML), adrenocortical carcinoma (ACC), bladder urothelial carcinoma (BLCA), breast invasive carcinoma (BRCA), cervical squamous cell carcinoma and endocervical adenocarcinoma (CESC), cholangiocarcinoma (CHOL), colon adenocarcinoma (COAD), esophageal carcinoma (ESCA), glioblastoma multiforme (GBM), head and neck squamous cell carcinoma (HNSC), kidney chromophobe (KICH), kidney renal clear cell carcinoma (KIRC), kidney renal papillary cell carcinoma (KIRP), lower grade glioma (LGG), liver hepatocellular carcinoma (LIHC), lung adenocarcinoma (LUAD), lung squamous cell carcinoma (LUSC), lymphoid neoplasm diffuse large B-cell lymphoma (DLBC), mesothelioma (MESO), ovarian serous cystadenocarcinoma (OV), pancreatic adenocarcinoma (PAAD), pheochromocytoma and paraganglioma (PCPG), prostate adenocarcinoma (PRAD), rectum adenocarcinoma (READ), sarcoma (SARC), skin cutaneous melanoma (SKCM), stomach adenocarcinoma (STAD), testicular germ cell tumors (TGCT), thymoma (THYM), thyroid carcinoma (THCA), uterine carcinosarcoma (UCS), uterine corpus endometrial carcinoma (UCEC), and uveal melanoma (UVM). Normal tissue: adipose tissue, adrenal gland, bladder, blood, blood vessel, brain, breast, cervix uteri, colon, esophagus, fallopian tube, heart, kidney, liver, lung, muscle, nerve, ovary, pancreas, pituitary, prostate, salivary gland, skin, small intestine, spleen, stomach, testis, thyroid, uterus, vagina.

### Differential expression analysis in TCGA datasets

In order to make more accurate results, we used paired tumor and normal samples to perform mRNA differential expression analysis. A total of 14 cancer types (BLCA, BRCA, COAD, ESCA, HNSC, KICH, KIRC, KIRP, LIHC, LUAD, LUSC, PRAD, STAD, and THCA) were included in the final analysis, which have over ten paired tumor and normal samples. The RNAseq by Expectation-Maximization (RSEM) values were utilized to quantify the mRNA expression levels. The fold change(FC) was calculated by mean(Tumor)/mean(Normal), the *P*-value was estimated by t-test and was further adjusted by false discovery rate (FDR). The genes with the threshold of FC > 2 and FDR ≤ 0.05 were considered as significantly differentially expressed.

### Subtype expression analysis and pathologic stage correlation

A great degree of intratumoral heterogeneity exists between tumors of different subtypes (molecular subtypes and clustering subtypes) in the same tumor type, which could be caused by different expression levels of genes in different subtypes of tumors. In order to find out the changes of gene expression related to subtypes, we performed expression subtype analysis. 9 cancer types (HNSC, LUSC, COAD, STAD, LUAD, GBM, BRCA, KIRC, BLCA), which have at least 10 samples of each subgroup in a subtype, were left for final analysis. We combined mRNA expression with clinical subtype data by matching the barcode of TCGA samples. We compared the gene expression of topoisomerase family genes among different subgroups in each subtype through the Wilcoxon test (number of subtype groups = 2) and ANOVA test (number of subtype groups > 2). Results were considered statistically significant at FDR ≤ 0.05. Furthermore, we performed tend analysis to explore the gene expression changes of topoisomerase family genes with the progression of clinicopathologic stage. 21 cancer types with at least 5 samples in each stage subgroup were incorporated into the final analysis. The pathologic stage classified samples into main stages I, II, III, and IV.

### Survival analysis based on gene expression levels

For expression survival analysis, we integrated the mRNA expression data of topoisomerase family genes with the corresponding clinical survival data by matching TCGA bar codes, leaving out some undeleted data. According to the median of RSEM standardized expression value, we divided the tumor samples into high expression group and low expression group. Then, we used the R package “survival” to fit the survival status and survival time within two groups. The Cox Proportional-Hazards model and log-rank test were performed for every gene in every cancer. The genes with *P*≤0.05 in Kaplan-Meier log-rank test were considered statistically significant.

### Methylation analysis

14 cancer types (THCA, KIRP, BLCA, LIHC, HNSC, BRCA, LUAD, PRAD, ESCA, KICH, LUSC, KIRC, STAD, COAD), which have more than 10 samples both in tumor and adjacent non-tumor tissues, were used to perform differential methylation analysis. The *P*-value was estimated by t-test and was further adjusted by FDR. Genes with FDR ≤0.05 were considered to have significant methylation differences.

The modification difference of DNA methylation can affect gene expression in theory. For correlation analysis between methylation levels and mRNA expression levels, we integrated mRNA expression data with methylation data by matching TCGA barcodes. Spearman correlation analysis was performed to get the correlation between matched mRNA expression and methylation levels. *P*-value was adjusted by FDR and genes with FDR ≤ 0.05 were considered to be influenced significantly by genome methylation.

Methylation data and clinical overall survival data were merged by sample barcode. Similar to expression survival analysis, the tumor samples were divided into high and low methylation groups according to the median methylation level. R package “survival” was used to fit survival time and survival status within two groups. Cox Proportional-Hazards model was constructed to get the risk ratio (Hazard ratio) of high methylation group compared with low methylation group. Log rank test was performed to test whether the survival difference between the two groups was statistically significant, and *P* ≤0.05 was considered as significant.

### SNV analysis

SNV analysis included 7 types of harmful mutations: Missense_Mutation, Nonsense_Mutation, Frame_Shift_Ins, Splice_Site, Frame_Shift_Del, In_Frame_Del, In_Frame_Ins. We generated the SNV summary diagram and the oncoplot waterfall diagram with the R package maftools. SNV percentage (frequency of deleterious mutations) of each gene’s coding region was calculated by the formula: Number of Mutated Samples/Number of Cancer Samples.

We integrated SNV data with clinical survival data by matching TCGA sample barcodes.Tumor samples were divided into mutant group when the specific gene was mutated (deleterious mutants). The log rank test was performed to test the survival difference between wild-type (WT) and Mutant groups.

### CNV analysis

In the CNV analysis, we calculated the percentage of CNV for topoisomerase family genes in different cancer types. CNV can be divided into two subtypes: heterozygous type and homozygous type, including amplified type and deletion type. The homozygote CNV represents CNV on both chromosomes, while the heterozygote CNV represents CNV on only one chromosome. The percentage statistic of CNV subtypes was based on CNV data processed by GISTICS 2.0.

For correlation analysis, we combined the mRNA expression data with the CNV raw data by matching the TCGA barcode. We calculated the association between matched mRNA expression and CNV percent samples based on Person’s product-moment correlation coefficient and t-distribution. *P*-value was adjusted by FDR.

For survival analysis, we combined CNV data with total clinical survival data by matching TCGA barcodes. The tumor samples were divided into wild-type, amplification and deletion groups. R package survival was used to fit survival time and survival status within groups. Log rank tests were performed to test the survival difference between the three groups, and *P* ≤0.05 was considered as significant.

### MicroRNA (miRNA) regulation network analysis

By matching TCGA barcode, we integrated the miRNA expression data and mRNA expression data. miRNA regulatory data includes experimental verification data (TarBase, miRTarBase and mir2disease) and predicted data (targetscan and miRanda). And only the miRNA-gene pairs who have been recorded in regulation data were used to calculate the expression correlation in all paired samples (33 cancers) based on Person’s product moment correlation coefficient and t-distribution. Considering the presence of positive regulators such as transcription factors, negatively related miRNA-gene pairs were considered as potential negative regulatory pairs. *P*-value was adjusted by FDR and genes with FDR≤ 0.05 and R<0 will be used to generate the network via visNetwork R packages.

### Pathway activity analysis

To obtain the relative protein levels, TCPA RPPA data were median-centered and normalized by standard deviation across all samples for each component. The pathway score is the sum of the relative protein levels of all positively regulated components of a given pathway minus the relative protein levels of the negatively regulated components. We calculated the pathway activity score (PAS) of 10 well-known cancer-related pathways (TSC/mTOR, RTK, RAS/MAPK, PI3K/AKT, Hormone ER, Hormone AR, EMT, DNA Damage Response, Cell Cycle, Apoptosis pathways). Tumor samples were divided into two groups (high and low) by median mRNA expression, and the PAS difference of the two groups was determined by the student t-test. When the PAS (gene X high group) > (gene X low group), we think that gene X may activate this pathway, otherwise it will inhibit it. PAS with FDR ≤ 0.05 was considered as significantly affecting the pathway.

### Immune subtype and drug sensitivity analysis

Immune subtype data were downloaded from UCSC Xena browser (http://xena.ucsc.edu/). In order to analyze the relationship between topoisomerase family gene expression and drug sensitivity, we integrated drug sensitivity data with mRNA expression data and of tumor cells. Pearson correlation analysis was performed to get the correlation between mRNA expression and drug IC50. *P*-value was adjusted by FDR. After integrating correlation coefficients and FDR, only the top 30 drugs were used for mapping.

### GSVA analysis

GSVA analysis estimated changes in gene set activity (expressed as topoisomerase scores) in the tumor sample population in an unsupervised manner. We calculated topoisomerase scores using the R package GSVA. This score is positively correlated with gene set expression and represents the comprehensive level of gene set expression. We assessed the abundance of immune cell infiltration using the ImmuCellAI web tool (http://bioinfo.life.hust.edu.cn/web/ImmuCellAI/). By integrating topoisomerase scores and corresponding data, we further explored the important role of topoisomerases in clinical staging, cancer subtypes, prognosis, immune cell infiltration, and pathway modulation.

### Statistical analysis

All statistical analyses were performed using the R software v3.6 (http://www.r-project.org) and SPSS version 23.0 (SPSS Inc, Chicago, IL, USA)

## Results

### mRNA expression and prognostic value of topoisomerase family genes

**Fig 1:**
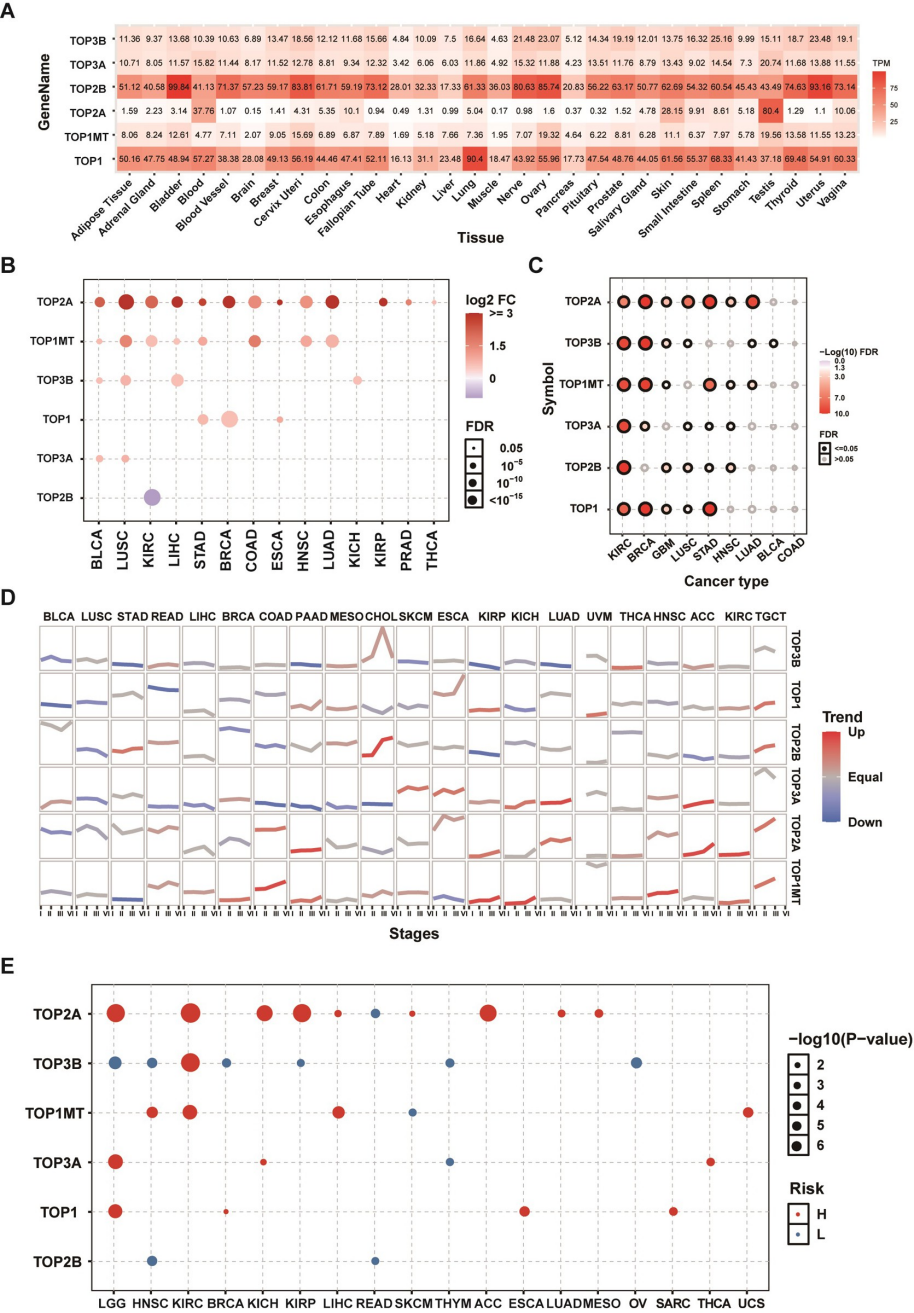
mRNA expression and survival analysis of topoisomerase family genes. (A) mRNA expression of topoisomerase family genes in the GTEx normal tissues. **(B)** Differential mRNA expression of topoisomerase family genes in paired tumor and paraneoplastic tissues. The bubble was filtered by the fold change (FC>2) and significance (FDR ≤0.05). **(C)** Differential expression of topoisomerase family genes associated with cancer subtypes. The black outline border of bubble indicates FDR≤0.05. **(D)** The trend of the gene expression of topoisomerase family genes from stage I to stage IV in different cancers. The blue trend line and red trend line represent fall and rise tendency, respectively. **(E)** Survival analysis of topoisomerase family genes in different types of cancer.

In order to investigate the gene expression of topoisomerase family genes in normal tissues, we extracted the mRNA expression levels of topoisomerase family genes in GTEx datasets. In all normal tissues, we found that the expression of TOP1 and TOP2B was overall higher relative to the expression of TOP3B, TOP3A, TOP2A and TOP1MT. Compared with other normal tissue, we found that the expression of TOP2B in bladder, cervix uteri, nerve, ovary and uterus; the expression of TOP2A in testis; the expression of TOP1 in lung were relatively high (Fig 1A). Subsequently, differential expression analysis was performed between tumor-normal paired samples from 14 TCGA cancer types. The results showed that the expressions of topoisomerase family genes were dysregulated in a variety of tumor types. The mRNA expressions of TOP2A in BLCA, LUSC, KIRC, LIHC, STAD, BRCA, COAD, ESCA, HNSC, LUAD, KIRP, PRAD and THCA; TOP1MT in BLCA, LUSC, KIRC, LIHC, STAD, COAD, HNSC and LUAD; TOP3B in BLCA, LUSC, LIHC and KICH; TOP1 in STAD, BRCA and ESCA; TOP3A in BLCA and LUSC were significantly upregulated. However, the mRNA expressions of TOP2B in KIRC was significantly downregulated (Fig 1B). It is noteworthy that different topoisomerase family genes may have an up- or down-regulation in the same types of cancer, such as TOP2A (S1A Fig) and TOP3B (S1B Fig) in KIRC. In order to determine whether the expression of topoisomerase family genes could affect the cancer subtype, we performed subtype expression analysis. The results showed that almost all topoisomerase family genes displayed a subtype-specific expression pattern in KIRC, BRCA, GBM, LUSC, STAD and HNSC (Fig 1C). For example, the expression of TOP2A was lower in luminal A and her2 subtype, especially in luminal A (S1C Fig), while the expression of TOP1 was lower in luminal A and basal subtype, especially in basal (S1D Fig). To test the role of topoisomerase family genes in tumor progression, we performed tend analysis in 21 TCGA cancer types with clinicopathologic information. We found that most topoisomerase family genes could have different effects on the progression of clinical stage in different cancer types (Fig 1D). It is noteworthy that the mRNA expressions of TOP2A and TOP1MT were upregulated progressively with advancing stage in many tumor types (Fig 1D). In addition, overall survival analysis showed that the expression of many topoisomerase family genes had differential effects on prognosis of cancer patients. The high expression of TOP2A in LGG, KIRC, KICH, KIRP, LIHC, SKCM, ACC, LUAD and MESP; TOP3B in KIRC; TOP1MT in HNSC, KIRC and LIHC; TOP3A in LGG, KICH and THCA; TOP1 in LGG, ESCA and SARC was associated with poor survival, while the high expression of TOP2A in READ; TOP3B in LGG, HNSC, BRCA, KIRP, THYM and OV; TOP1MT in SKCM; TOP3A in THYM; TOP2B in HNSC and READ was associated with good survival (Fig 1E). It is noteworthy that the mRNA expression of the same topoisomerase family genes could have different impacts on the prognosis in different cancer types. As an example, LGG patients with high TOP2A expression had poor survival (S1E Fig), while the high expression of TOP2A was associated with good survival in READ (S1F Fig). The results of these studies indicated that topoisomerase family genes might have an important role in tumorigenesis, cancer progression and intratumoral heterogeneity.

### Methylation analysis of topoisomerase family genes

**Fig 2:**
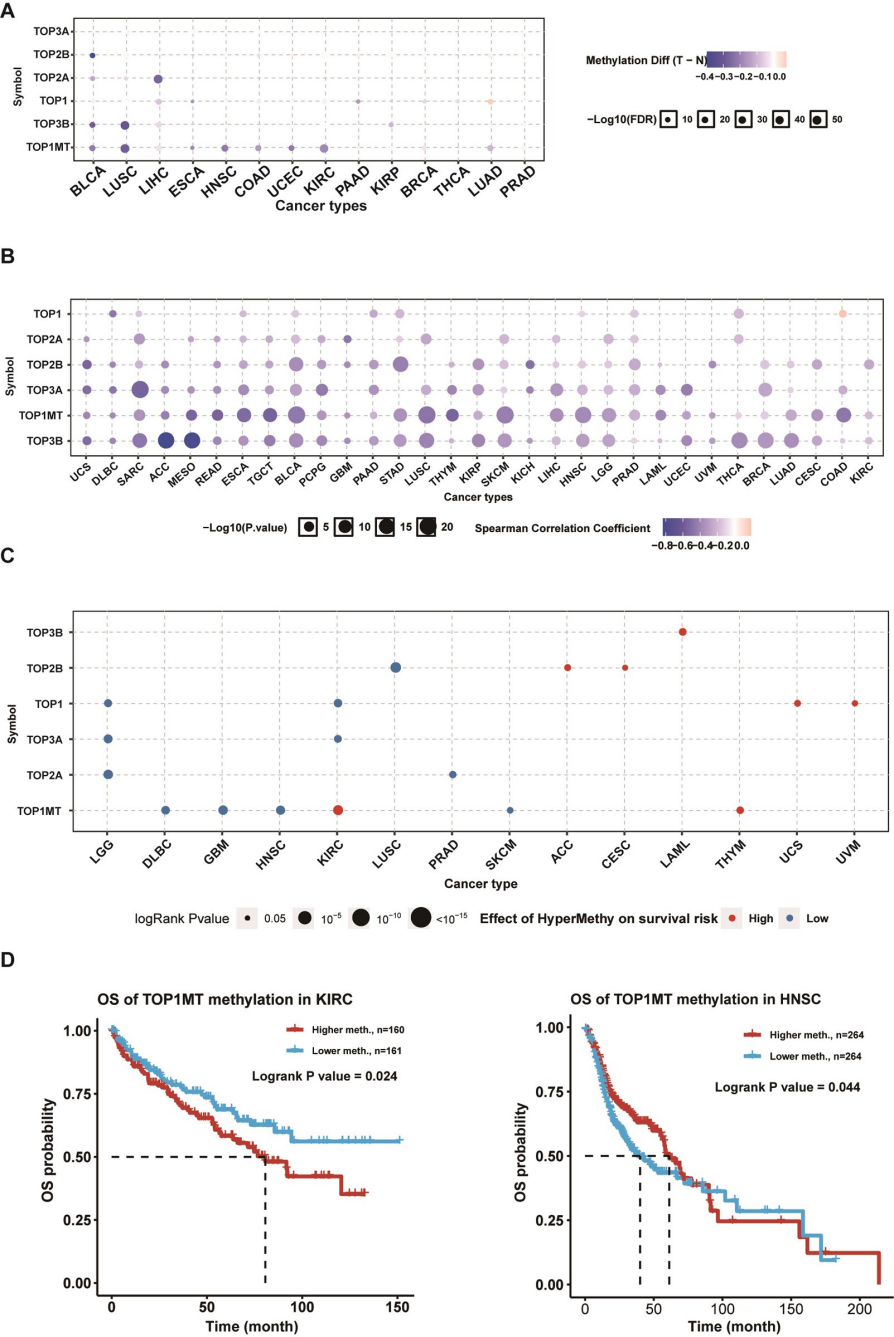
Methylation analysis of topoisomerase family genes. **(A)** Differential methylation status of topoisomerase family genes in paraneoplastic and tumor tissues in different cancer types. The bubble size is positively correlated with the FDR significance, and the bubble was filtered by FDR significance (FDR ≤0.05). **(B)** Relationship between methylation level and mRNA expression. **(C)** Analysis of methylation survival of topoisomerase family genes in different cancers. **(D)** Survival curves between hypermethylation and hypomethylation groups of TOP1MT in KIRC and HNSC.

To assess the corresponding differences in epigenetic regulation, we studied the methylation status of topoisomerase family genes between tumor and normal samples from 14 TCGA cancer types. We found that most topoisomerase family genes, such TOP1MT, TOP3B, TOP1 and TOP2A, exhibited DNA hypomethylation in multiple tumors (Fig 2A). Correlation analysis indicated that the DNA methylation negatively correlated with the expression of almost all topoisomerase family genes in pan-cancer (Fig 2B). Prognosis analysis indicated that the hypermethylation of TOP3B in LAML; TOP2B in ACC and CESC; TOP1 in UCS and UVM, TOP1MT in KIRC and THYM was associated with a poor prognosis, while the hypermethylation of TOP2B in LUSC; TOP1 in LGG and KIRC; TOP3A in LGG and KIRC; TOP2A in LGG and PRAD; TOP1MT in DLBC, GBM, HNSC and SKCM was associated with good prognosis (Fig 2C). It is noteworthy that there is a possibility that hypermethylation of the same topoisomerase family gene could have different effects on prognosis in different cancer types, such as TOP1MT in KIRC and HNSC (Fig 2D). These results suggested that the epigenetic modification patterns of genes from the topoisomerase family were abnormal in multiple tumors, influencing gene expression and prognosis in patients with cancer.

### Mutational landscape of topoisomerase family genes

**Fig 3:**
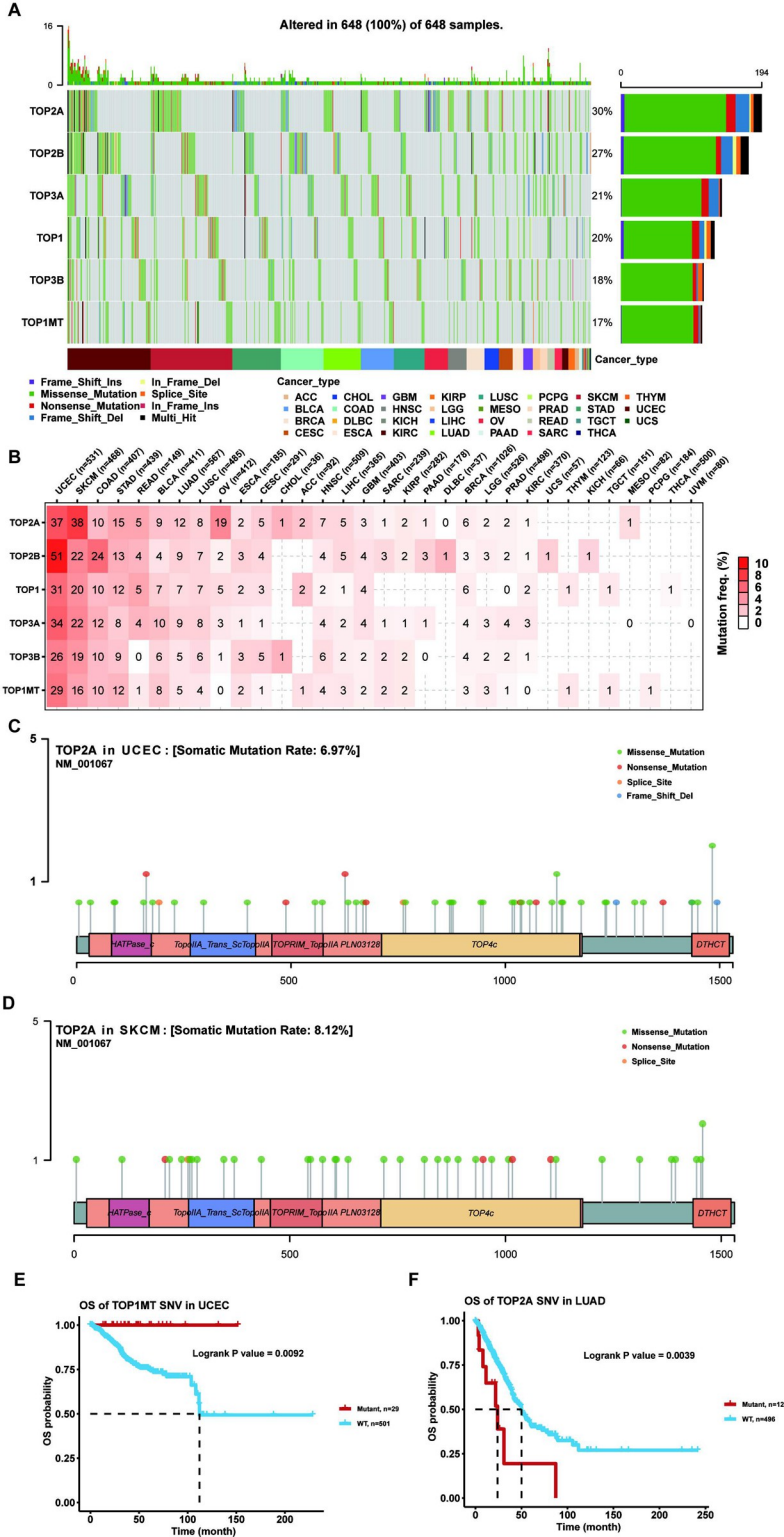
SNV analysis of topoisomerase family genes. **(A)** The waterfall diagram showed the SNV frequency distribution of topoisomerase family genes in different types of tumors. Side barplot and top barplot show the number of variants in each gene and each sample, respectively. **(B)** Percentage heatmap showed the topoisomerase family genes SNV frequency in different types of cancer. Each unit of the number represents the number of samples in certain types of cancer mutations. The 0 and blank in the cell indicate there is no mutation in specific gene coding region and all regions of a specific gene, respectively. **(C)** Lollipop diagrams of TOP2A mutation sites, types and frequencies in UCEC. **(D)** Lollipop diagrams of TOP2A mutation sites, types and frequencies in SKCM. **(E)** Survival curve between WT and Mutant groups of TOP1MT in UCEC. **(F)** Survival curve between WT and Mutant groups of TOP2A in LUAD.

The occurrence and development of cancer is related to somatic mutations in cancer genomes. Thus, our study examined SNP data for topoisomerase family genes in pan-cancer. The waterfall plot showed that the mutation frequencies of TOP2A, TOP2B, TOP3A, TOP1, TOP3B and TOP1MT were 30%, 27%, 21%, 20%, 18% and 17%, respectively (Fig 3A). In addition, the SNV summary plot showed that the most common SNV class (base substitution) was C>T, while the most prevalent variant types were missense mutations and SNPs. (S2 Fig). In the SNV percentage analysis, we found that the number of samples in which the topoisomerase family genes occurred deleterious mutation was greater in UCEC and SKCM, such as the TOP2A with the highest mutation frequency among all cancer types (Fig 3B–3D). Survival analysis found that the mutations in different genes of the topoisomerase family may have differential effects on cancer prognosis (data not shown). For example, the TOP1MT mutation was associated with better survival in UCEC (Fig 3E), while the TOP2A mutation was associated with poor survival in LUAD (Fig 3F). These results indicated that genes in the topoisomerase family were particularly prone to mutation in tumors, and their mutations could also affect cancer patient outcomes.

### CNV of topoisomerase family genes

**Fig 4:**
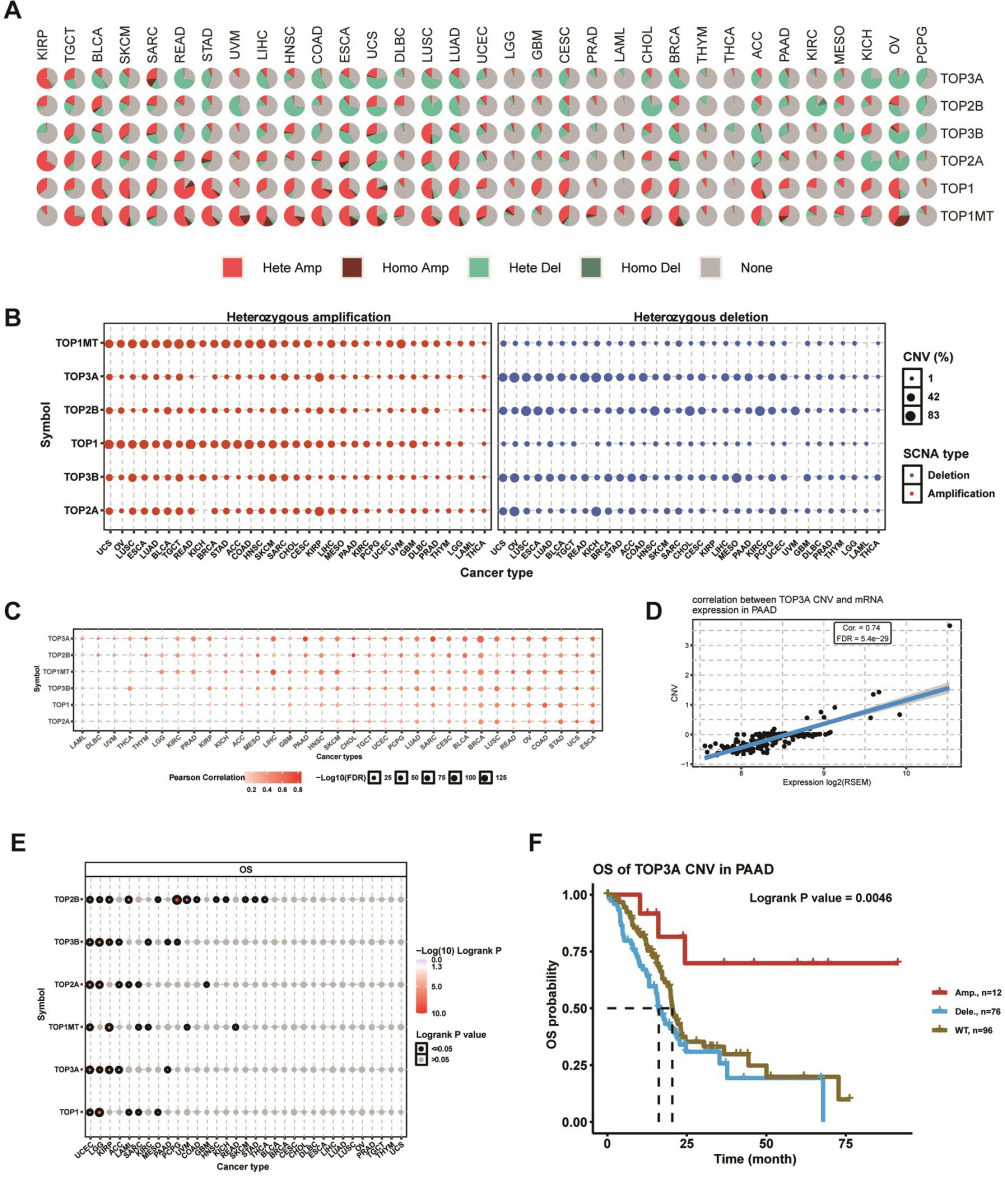
CNV analysis of topoisomerase family genes. **(A)** CNV pie chart showed the composition of heterozygous / homozygous CNV of topoisomerase family genes in different cancer types. **(B)** The heterozygous CNV diagram showed the percentage of heterozygous amplification (red bubbles) and deletions (blue bubbles) of topoisomerase family genes in different types of cancer. The bubble size positively correlated with percentage. **(C)** Correlation between CNV level and gene expression of topoisomerase family in different types of cancer. **(D)** Scatter diagram showed the relationship between TOP3A CNV and its mRNA expression in PAAD. **(E)** CNV survival analysis of topoisomerase family genes in different types of cancer. **(F)** Kaplan-Meier curve showing the survival difference between different CNV types and wild type of TOP3A in PAAD.

The presence of CNV is a common and important hallmark of many cancers. In light of this, we investigated the CNV changes of topoisomerase family genes in further depth. As shown in the CNV pie plot, the vast majority of topoisomerase family genes occurred large number of copy number amplifications and deletions in most cancer types (Fig 4A). Among them, TOP1 in READ had the highest relative percentage of total amplifications, while TOP2B in KIRC had the highest relative percentage of total deletions (S3A Fig). In addition, heterozygous CNV analysis showed that almost all topoisomerase family genes had heterozygous amplification and deletion among all cancer types (Fig 4B). Meanwhile, homozygous CNV analysis showed that TOP1MT in 31 cancer types, TOP2A in 20 cancer types, TOP1 in 19 cancer types, TOP3B in 21 cancer types, TOP3A in 14 cancer types, and TOP2B in 17 cancer types occurred homozygous amplifications, while TOP1MT in 14 cancer types, TOP2A in 14 cancer types, TOP1 in 9 cancer types, TOP3B in 20 cancer types, TOP3A in 18 cancer types, and TOP2B in 14 cancer types occurred homozygous deletions (S3B Fig). Correlation analysis indicated that the mRNA expressions of topoisomerase family genes were positively correlated with their copy number levels in most cancers, such as TOP3A in PAAD and TOP2A in UCEC (Figs 4C and 4D and S3C). Survival analysis showed that the CNVs of TOP2B in 13 cancer types, TOP3B in 7 cancer types, TOP2A in 6 cancer types, TOP1MT in 6 cancer types, TOP3A in 5 cancer types and TOP1 in 5 cancer types significantly correlated with overall prognosis (Fig 4E). For example, the copy number amplifications of TOP3A in PAAD associated with better prognosis compared with normal copy number and copy number deletions (Fig 4F), while the copy number amplifications and deletions of TOP2A in UCEC were associated with poor prognosis compared with normal copy number (S3D Fig). Noteworthy is the fact that the CNV of all topoisomerase family genes could affect the prognosis of patients with UCEC (Fig 4E). These results indicated that different types of CNV of topoisomerase family genes were prevalent in many types of cancer and could affect the cancer patients’ mRNA expression and prognosis.

### miRNA regulation of topoisomerase family genes

**Fig 5:**
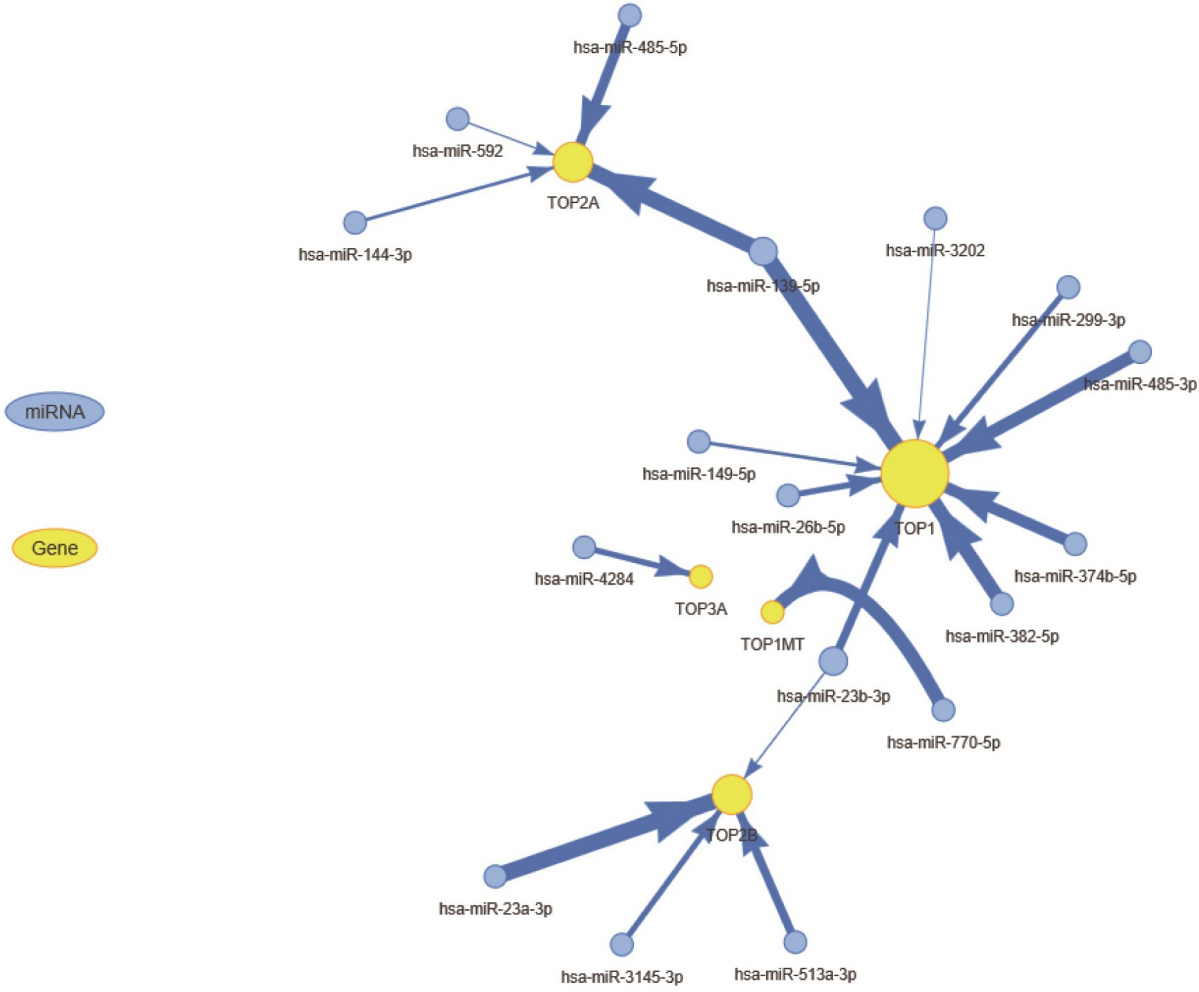
The miRNA regulation network of topoisomerase family genes. The association between miRNAs and genes suggests that miRNAs have regulatory effects on genes. The size of the node and the degree of the node are positively correlated, and the width of the line is determined by the absolute value of the correlation coefficient.

miRNA-mediated regulation of cancer-related genes play an important role in cancer development and progression. In order to clarify which miRNAs are responsible for regulating mRNA expression of topoisomerase family genes, we used R package visNetwork to construct the miRNA-gene regulation networks (Fig 5). In this network, node size of gene and edge width were positively correlated to the number of related miRNAs and correlation coefficient respectively. We found that TOP1, TOP2A and TOP2B were negatively regulated by more miRNAs. It is noteworthy that some topoisomerase family genes could be regulated by multiple miRNAs. For instance, both of TOP1 and TOP2A can be negatively regulated by hsa-miR-139-5p. Based on these results, we found it appears that the mRNA expressions of topoisomerase family genes were subject to complex miRNA regulatory networks.

### Pathway activity analysis

**Fig 6:**
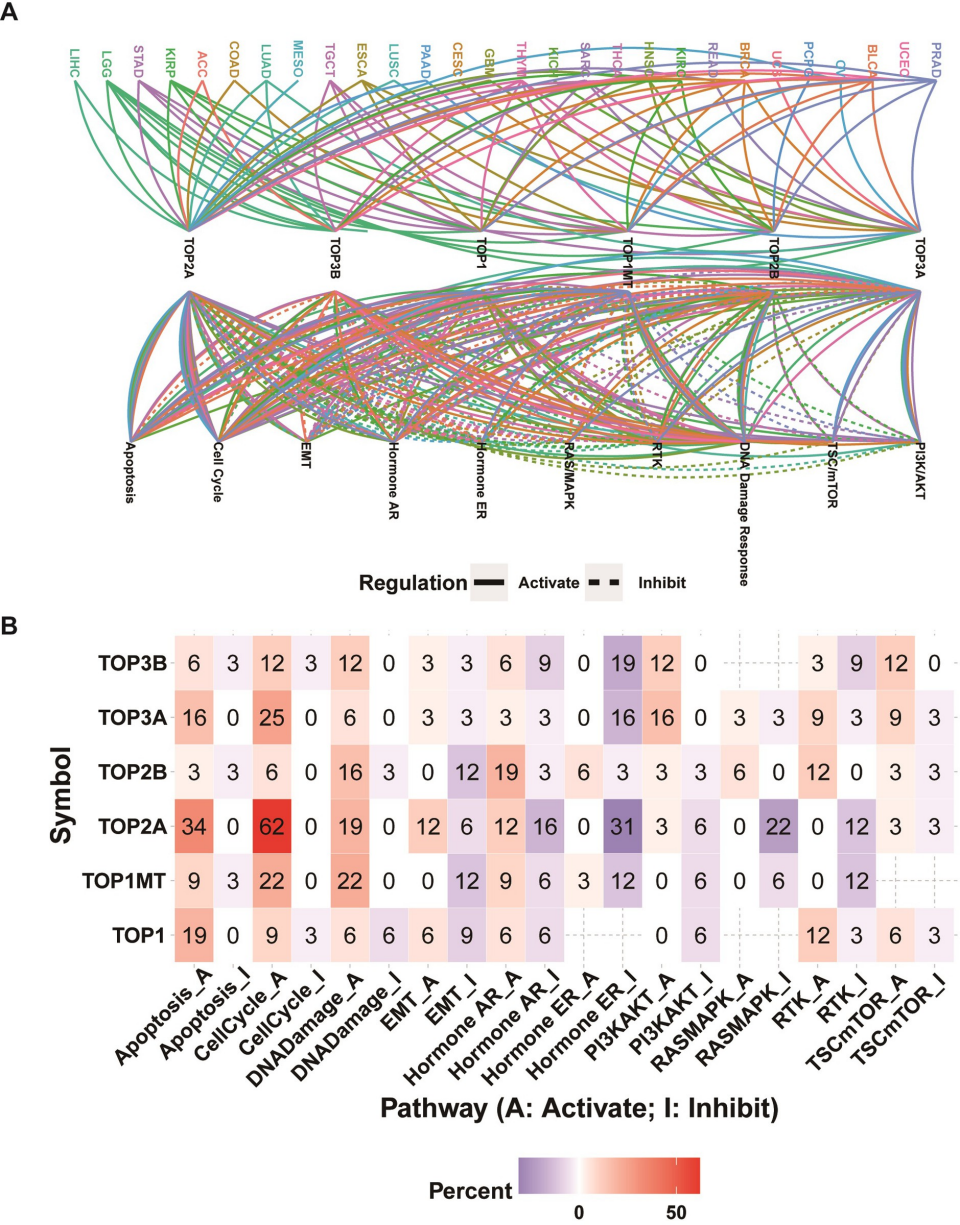
Pathway activity analysis of topoisomerase family genes. **(A)** Gene-pathway network showed the regulatory relationship between topoisomerase family genes and tumor pathways in pan-cancer. **(B)** Heatmap showing the percentage of cancer types in which specific topoisomerase family genes have an effect on specific pathways (FDR ≤ 0.05) in pan-cancer.

The global regulation network showed that the mRNA expression of topoisomerase family genes had intricate connections with the cancer-related pathway activity in pan-cancer (Fig 6A). Noteworthy is the fact that the same topoisomerase family genes may produce different regulatory effects on the same pathway in different cancer types. For example, a high level of TOP2A expression could promote the activation. As an example, high expression of TOP2A could promote the activation of EMT pathway in LIHC whereas inhibiting the activation of EMT pathway in STAD (S4A and S4B Fig). The pathway heatmap and pie chart showed the percentage of cancer types (cancer types/32 *100%) which topoisomerase family genes affected the specific pathway (Figs 6B and S4C). Apoptosis pathway was predominantly activated by TOP3B (6% activation vs. 3% inhibition), TOP3A (16% activation vs. 0% inhibition), TOP2A (34% activation vs. 0% inhibition), TOP1MT (9% activation vs. 3% inhibition), TOP1 (19% activation vs. 0% inhibition). Similarly, all topoisomerase family genes could activate the cell-cycle pathway. Except for TOP1, all other topoisomerase family genes could activate the DNA damage pathway. EMT pathway was predominantly activated by TOP2A and inhibited by TOP2B, TOP1 and TOP1MT. Hormone AR pathway was predominantly activated by TOP2B and TOP1MT and inhibited by TOP2A. Hormone ER pathway was predominantly activated by TOP2B and inhibited by TOP3B, TOP3A, TOP2A and TOP1MT. PI3K/AKT pathway was predominantly activated by TOP3B and TOP3A and inhibited by TOP2A, TOP1MT and TOP1. RAS/MAPK pathway could be activated by TOP2B and inhibited by TOP2A and TOP1MT. RTK pathway was predominantly activated by TOP3A, TOP2B and TOP1 and inhibited by TOP3B, TOP2A and TOP1MT. TSC/mTOR pathway could be activated by TOP3B, TOP3A and TOP1 (Fig 6B). These results suggested that topoisomerase family genes contributed greatly in mediating the inhibition or activation of cancer-related pathways.

### Immune subtype and drug sensitivity analysis

**Fig 7:**
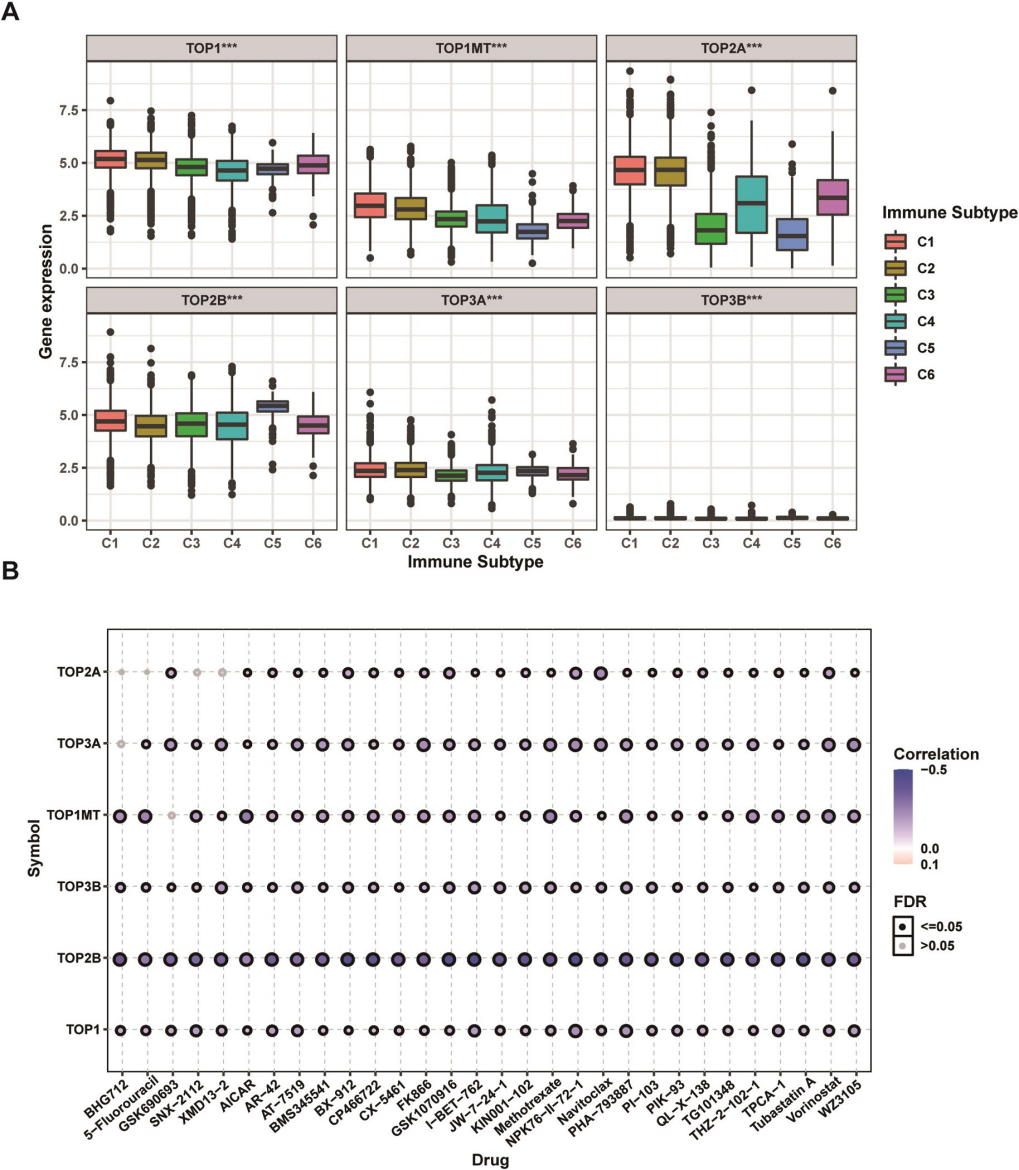
Immune subtype and drug sensitivity analysis of topoisomerase family genes. **(A)** Differential expression of topoisomerase family genes in six pan-cancer immune subtypes. **(B)** Bubble diagram of the relationship between drug sensitivity (IC50) and gene expression level of topoisomerase family genes in CTRP database. Positive correlation (red bubble) indicates one gene with high expression was resistant to a drug, while negative correlation (blue bubble) indicates one gene with high expression was sensitive to a drug. The color depth and size of bubble are positively correlated with the correlation coefficient and the FDR significance, respectively. Black outline border indicates FDR≤0.05.

Extensive genomic alterations may affect cancer patients’ immune responses and anticancer drug sensitivity. Based on the results of previous sections, thus, we further explored in depth whether the topoisomerase family genes would affect the immune status and drug sensitivity of patients with cancer. Immune subtype analysis showed that all topoisomerase family genes were differentially expressed in different immune subtypes (Fig 7A). It is noteworthy that, except for TOP2B, all other opoisomerase family genes were found to be highly expressed in C1 and C2 subtype (Fig 7A). Subsequently, drug correlation analysis showed that the mRNA expressions of almost all topoisomerase family genes were mainly positively correlated with the sensitivity of top 30 ranked drugs (negtive correlation with IC50) (Fig 7B). It is noteworthy that the mRNA expressions of TOP3B presented the strongest and most significant correlation. These results revealed that the dysregulated expression of topoisomerase family genes could have close connections to the microenvironment of tumor immunity and impact the response of anticancer treatment.

### GSVA analysis

**Fig 8:**
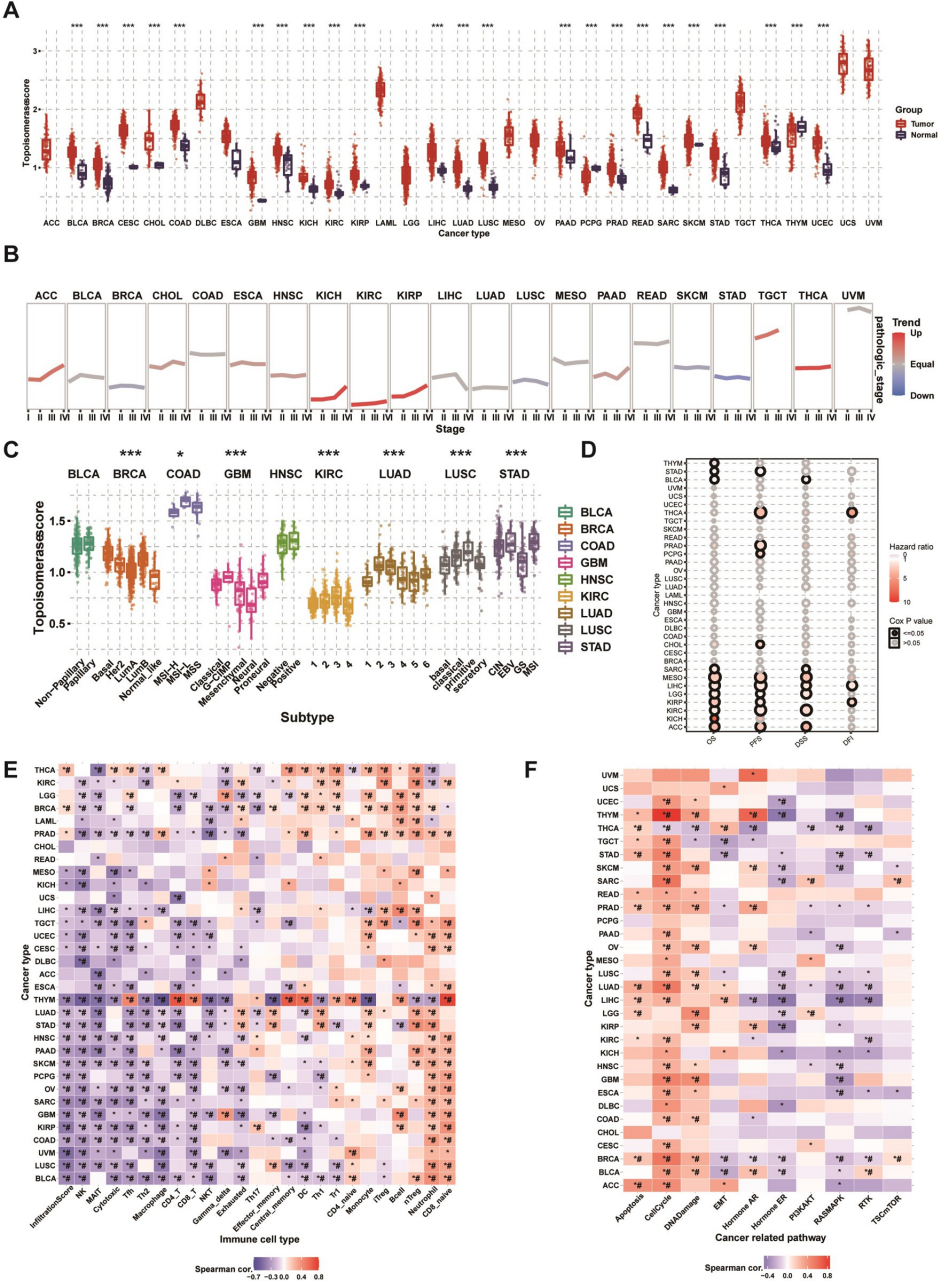
GSVA analysis of topoisomerase family genes. **(A)** Differences of topoisomerase score in paraneoplastic and tumor tissues. The topoisomerase score represents the integrated level of the expression of topoisomerase family genes, which is positively correlated with gene expression. **(B)** Trend of topoisomerase scores from stage I to stage IV tumors. The blue trend line represents a decreasing score and the red trend line represents an increasing score. **(C)** Differences in topoisomerase scores among different cancer subtypes. **(D)** Survival analysis of topoisomerase score in different cancer types, including OS, PFS, DSS, and DFI. **(E)** Correlation heatmap between topoisomerase score and immune cell infiltration in different cancer types. *: P value≤0.05; #: FDR≤0.05. **(F)** Correlation heatmap between topoisomerase score and pathway activity in different cancer types. *: P value≤0.05; #: FDR≤0.05.

Based on encouraging results described in previous sections, we used GSVA method to further assess relative activation levels of topoisomerase in pan-cancer. Differential expression analysis indicated that the topoisomerase score was upregulated in tumor tissues (Fig 8A). Pathologic stage tend analysis displayed that the topoisomerase score was upregulated progressively with advancing stage in many tumor types, including ACC, KICH, KIRC, KIRP, PAAD, TGCT and THCA (Fig 8B). Subtype analysis showed that the topoisomerase scores were significantly different in different subtypes of multiple tumors, including BRCA, COAD, GBM, KIRC, LUAD, LUSC and STAD (Fig 8C). Survival analysis displayed that the topoisomerase score could affect the OS of THYM, STAD, BLCA, SARC, MESO, LIHC, LGG, KIRP, KIRC, KICH and ACC, the PFS of STAD, THCA, PRAD, PCPG, CHOL, MESO, LIHC, LGG, KIRP, KIRC and ACC, the DSS of BLCA, SARC, MESO, LIHC, LGG, KIRC and ACC and the DFI of THCA, LIHC and KIRP (Fig 8D). It is noteworthy that the topoisomerase score could affect more than one type of survival in some cancer types. For instance, patients with lower topoisomerase scores had longer overall survival (OS), progression-free survival (PFS), disease-specific survival (DSS), and disease-free survival (DFI) in LIHC (S5A–S5D Fig). In addition, surprisingly, the results of immune infiltration analysis were generally consistent in different cancer types (Fig 8E). We found that the topoisomerase score was positively correlated with the cell abundance of CD8_naive, neutrophil, nTreg, B cell, iTreg, monocyte and CD4_naive. On the contrary, the topoisomerase score was negatively correlated with the cell abundance of NK, MAIT, Tfh, Th2, macrophage, CD4_T, CD8_T, NKT, gamma delta, exhausted. It should be paid particular attention that the topoisomerase score of almost all types of cancer was negatively correlated with the cytotoxic cell abundance and infiltration score. Subsequently, we performed pathway activity analysis to explore the intrinsic interactions between the topoisomerase score and canonical cancer-related pathways (Fig 8F). In the vast majority of tumors, the topoisomerase score correlated positively with the pathway activation of apoptosis, cell cycle, DNA damage. In contrast, the topoisomerase score negatively correlated with the pathway activation of hormone ER, RAS/MAPK and RTK. These results confirmed again that topoisomerase could play important roles in malignancies by affecting the tumor progression, subtype heterogeneity, recruitment of immune cells, regulation of oncogenic pathway and the cancer patients’ prognosis.

## Discussion

Topoisomerases play important roles in cell proliferation, differentiation and other life processes, and their expression level increase in multiple solid tumors [14–17]. More and more studies have shown that dysregulation of topoisomerases associated with many human diseases including tumors [18–20]. Selective targeting topoisomerase has always been a hot topic in anticancer therapy [21,22]. Thus, it has a great deal of significance to study the roles of topoisomerase family genes in tumorigenesis and cancer progression. In this study, we performed a systematic integrative investigation of 6 topoisomerase family genes across 33 cancer types by using pan-cancer multiomics data. Our results indicated that alterations to the genome, abnormal epigenetic modifications and complex regulation of miRNAs of topoisomerase family genes resulted in their dysregulated gene expression, which correlated significantly with the changes of immune microenvironment, disturbed regulation of cancer hallmark-related pathways and clinical prognosis. According to our knowledge, this is the first report of topoisomerase family genes based on a pan-cancer perspective.

In gene expression and survival analysis, we found that the topoisomerase family genes exhibited varying degrees of expression dysregulation in multiple cancers, which in turn could impact the progression of clinical stage, subtype heterogeneity and final outcomes of patients with cancer. As an extensive differentially expressed gene in 13 cancer types, a high level of TOP2A expression has been indicated in multiple cancers, including high-grade serous ovarian cancer, hepatocellular carcinoma, esophageal squamous cell carcinoma [23–25]. In addition, patients with TOP2A high expression showed a significantly higher rate of distant metastasis in early stage luminal breast cancer [26]. Another two study showed that high expression of TOP2A was associated with poor prognosis of non-small cell lung cancer and clear cell renal cell carcinoma [27,28]. These studies described above are consistent with our findings. Nevertheless, there was also non-conformity between aberrant gene expression and clinical prognosis. For instance, the mRNA expression of TOP2A was not different in KICH; however, high expression of TOP2A was associated with poor survival in KICH. Hence, we speculated that topoisomerase family genes would possibly occur genetic modification alterations in neoplasm progression.

In epigenetic and genome analysis, we found that topoisomerase family genes had extremely complex genomes with abnormal epigenetic modification patterns, high mutation frequency and extensive copy number alterations in pan-cancer. These changes mediated the transcriptional dysregulation of topoisomerase family genes and dramatically altered cancer prognosis. A recent study reported the 5Aza (DNA demethylation agents) pre-treatment could induce the increased expression of TOP1MT and the impacxt the death, growth and differentiation of glioblastoma cell lines(HSR-GBM1) [29]. Another study showed that the DNA methylation of TOP2B has a typical male bias in bladder cancer, and its related drug valrubicin can be used for intravesical treatment of BCG-refractory bladder carcinoma in situ [30]. In addition, as the highest gene with mutational frequency in pan-can, TOP2A mutations have been proven to correlate with cancer progression and drug resistance in a variety of tumor types, including breast cancer [31], glioblastoma [32]and cervical cancer [33]. Another study revealed that copy number gains of TOP1 was found in colorectal cancers, which could affect the drug sensitivity of irinotecan and anthracycline [34]. The results of these studies were basically consistent with our study. In the miRNA-mRNA interaction network analysis, we found that the miRNAs which could negatively regulate the topoisomerase family genes. Multiple studies have shown the miRNAs may negatively regulate topoisomerase family genes and play necessary roles within the management of cancer progression and metastasis [35–37]. These results indicated that miRNA played critical roles in regulating topoisomerase family genes, which might be concerned in inhibiting tumour progression and up survival in numerous cancers.

In the pathways analysis, we found the topoisomerase family genes were involved in regulation of cancer-related signaling pathways. Taken as a whole, topoisomerase family genes could activate apoptosis, cell cycle, DNA damage and inhibit hormone ER, RAS/MAPK and RTK which have been verified in several cancers [38–42]. There is growing evidence that topoisomerase had an intricate relationship with immune response and drug sensitivity [43–46]. In immune subtype analysis, we found that the mRNA expression of topoisomerase family genes differed greatly in different immune subtypes. In drug sensitivity analysis, we screened potential anticancer drugs specifically targeting the topoisomerase family genes. McKenzie et al. reported that TOP1 inhibitors can increase the sensitivity of patient-derived melanoma cell lines to T-cell-mediated cytotoxicity and improve the antitumor efficacy of cancer immunotherapy [47]. In addition, Burgess et al. reported that the expression levels of topoisomerase family members were the major determinants of chemotherapy response of multiple drugs [48]. Finally, we again confirmed that topoisomerase was closely related to tumorigenesis, cancer progression, intratumoral heterogeneity, immune-active microenvironment, regulation of cancer-related pathways and final clinical outcomes through GSVA analysis.

## Conclusion

In conclusion, we have performed a multi-omics pan-cancer analysis of topoisomerase family genes, which will help reveal their potential molecular mechanisms of oncogenesis and provide new clues for precise diagnosis and personalized treatment of cancer.

## Supporting information

S1 Fig (A) Differential expression of TOP2A in paraneoplastic tissues and KIRC. (B) Differential expression of TOP2B in paraneoplastic tissues and KIRC. (C) Differential expression of TOP2A in different BRCA subtypes. (D) Differential expression of TOP1 in different BRCA subtypes. (E) Kaplan-Meier survival curves for TOP2A in LGG. (F) Kaplan-Meier survival curves for TOP2A in READ.
(TIF)

S2 Fig An overview of SNV showing the number and type of mutations. (TIF)

S3 Fig (A) CNV type proportion of TOP1 in READ and TOP2B in KIRC. (B) Homozygous CNV diagram showing the proportion of homozygous amplification and deletion of topoisomerase family genes in different cancers. (C) Scatter plot showing the correlation between TOP2A CNV and its mRNA expression in UCEC. (D) Kaplan-Meier curve showing the survival difference between different CNV types and wild type of TOP2A in UCEC.
(TIF)

S4 Fig (A) The box chart showed the difference of EMT pathway activity score between TOP2A high expression group and TOP2A low expression group in LIHC. (B) The box chart showed the difference of EMT pathway activity score between TOP2A high expression group and TOP2A low expression group in STAD. (C) Pathway pie plot showing the global percentage of cancer types in which the specific topoisomerase family genes has an effect on the specific pathway in pan-cancer.
(TIF)

S5 Fig Kaplan-Meier curve showing the survival difference between high and low topoisomerase score in LIHC, including OS (A), PFS (B), DSS (C) and DFI (D).
(TIF)

S1 File Data and code. (ZIP)
