# Supplementary figures and images for: An integrated multi-omics analysis of topoisomerase family in pan-cancer: Friend or foe?
Source: PLoS One. 2022 Oct 26;17(10):e0274546. doi: 10.1371/journal.pone.0274546 (PMC9604985; doi:10.1371/journal.pone.0274546)

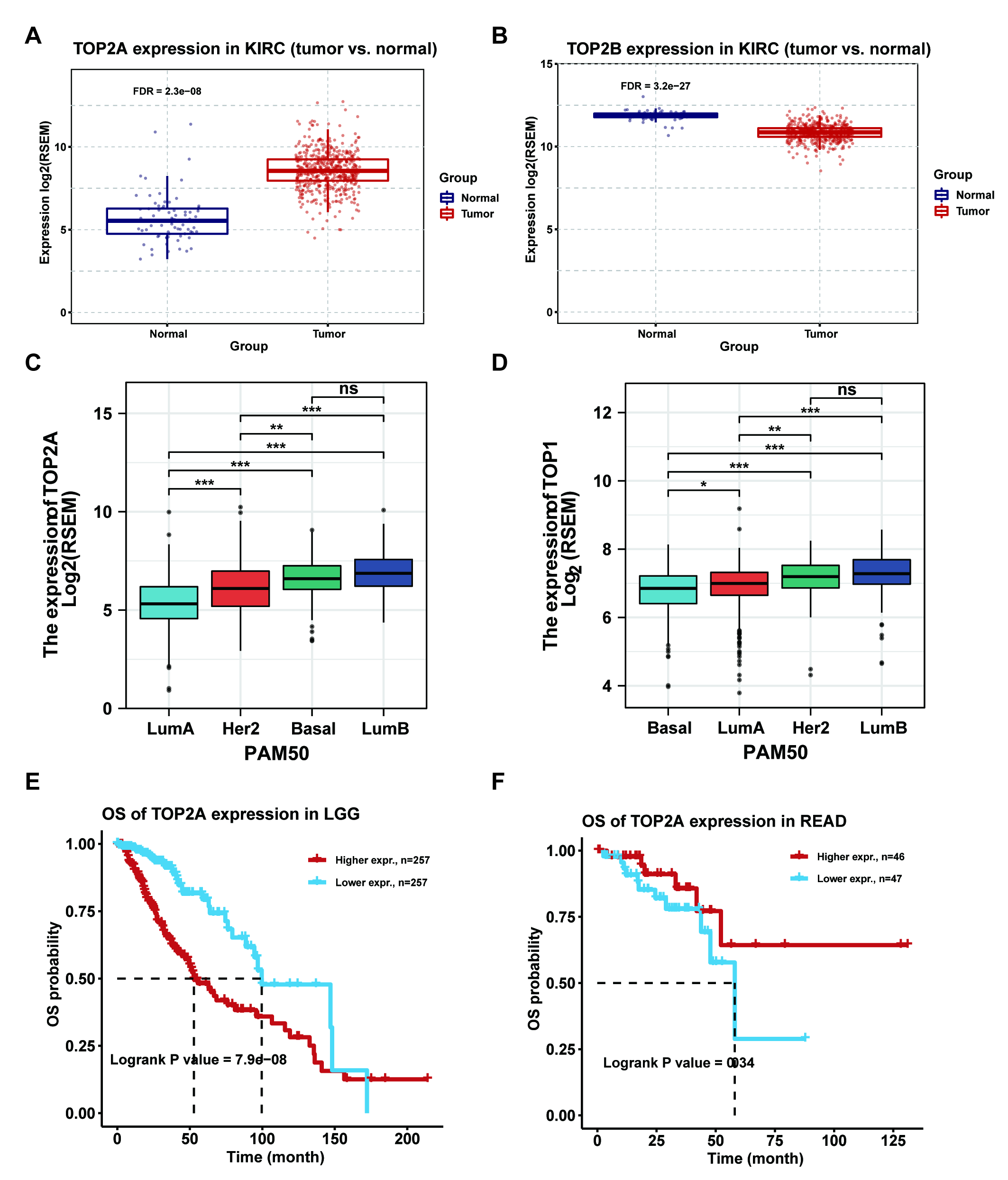

Supplement: S1 Fig — (A) Differential expression of TOP2A in paraneoplastic tissues and KIRC. (B) Differential expression of TOP2B in paraneoplastic tissues and KIRC. (C) Differential expression of TOP2A in different BRCA subtypes. (D) Differential expression of TOP1 in different BRCA subtypes. (E) Kaplan-Meier survival curves for TOP2A in LGG. (F) Kaplan-Meier survival curves for TOP2A in READ. (TIF) [file pone.0274546.s001.tif]

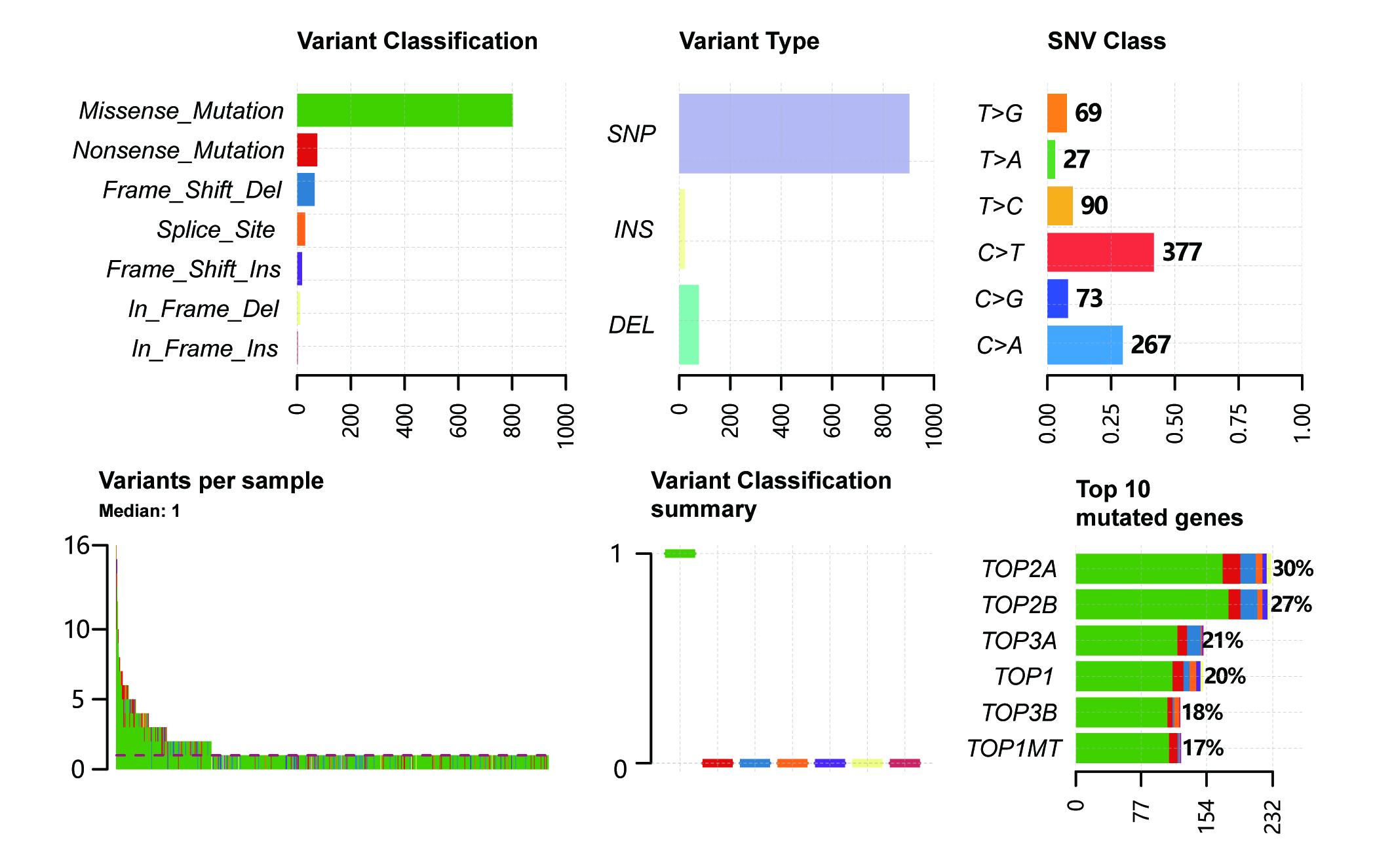

Supplement: S2 Fig — (TIF) [file pone.0274546.s002.tif]

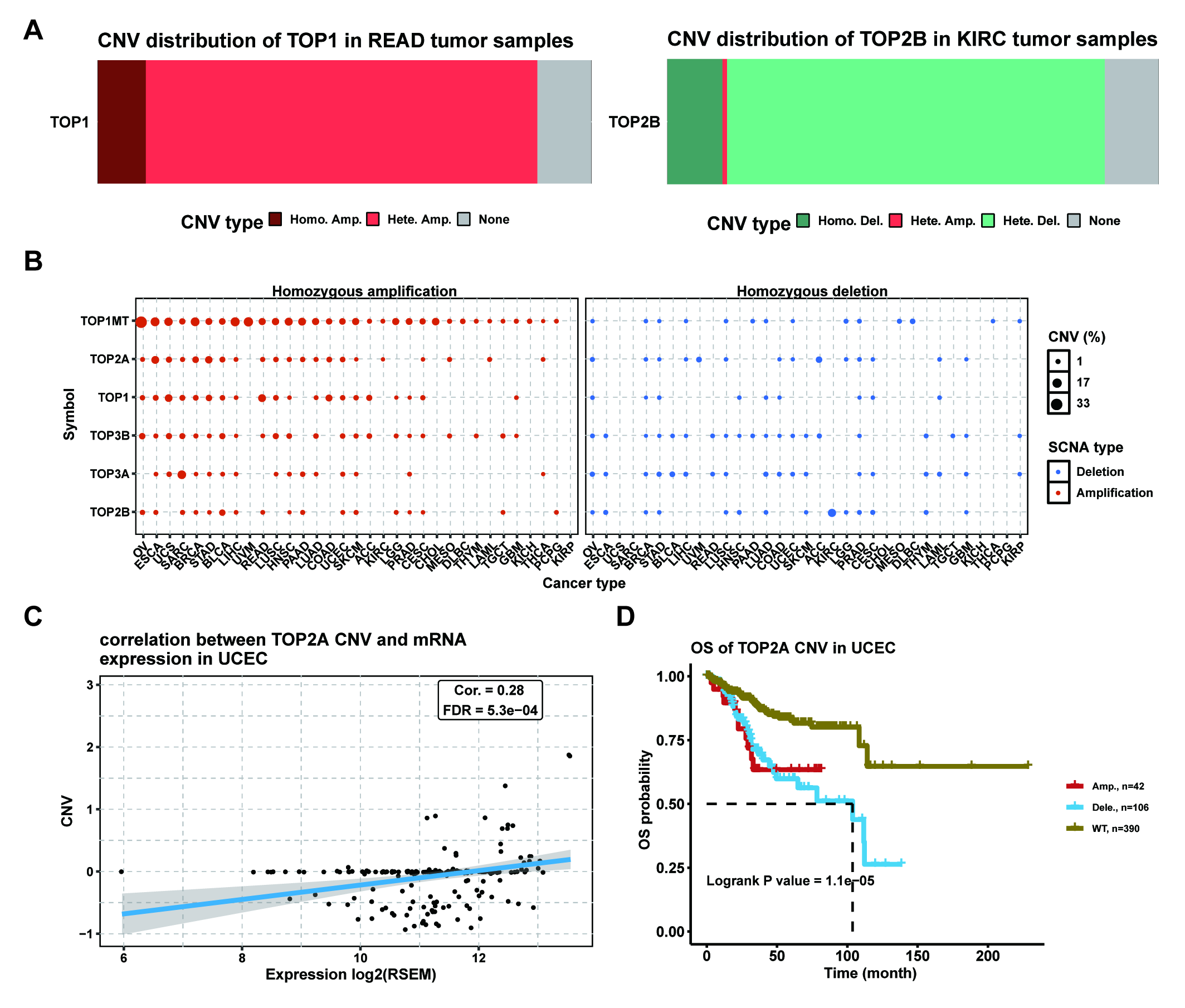

Supplement: S3 Fig — (A) CNV type proportion of TOP1 in READ and TOP2B in KIRC. (B) Homozygous CNV diagram showing the proportion of homozygous amplification and deletion of topoisomerase family genes in different cancers. (C) Scatter plot showing the correlation between TOP2A CNV and its mRNA expression in UCEC. (D) Kaplan-Meier curve showing the survival difference between different CNV types and wild type of TOP2A in UCEC. (TIF) [file pone.0274546.s003.tif]

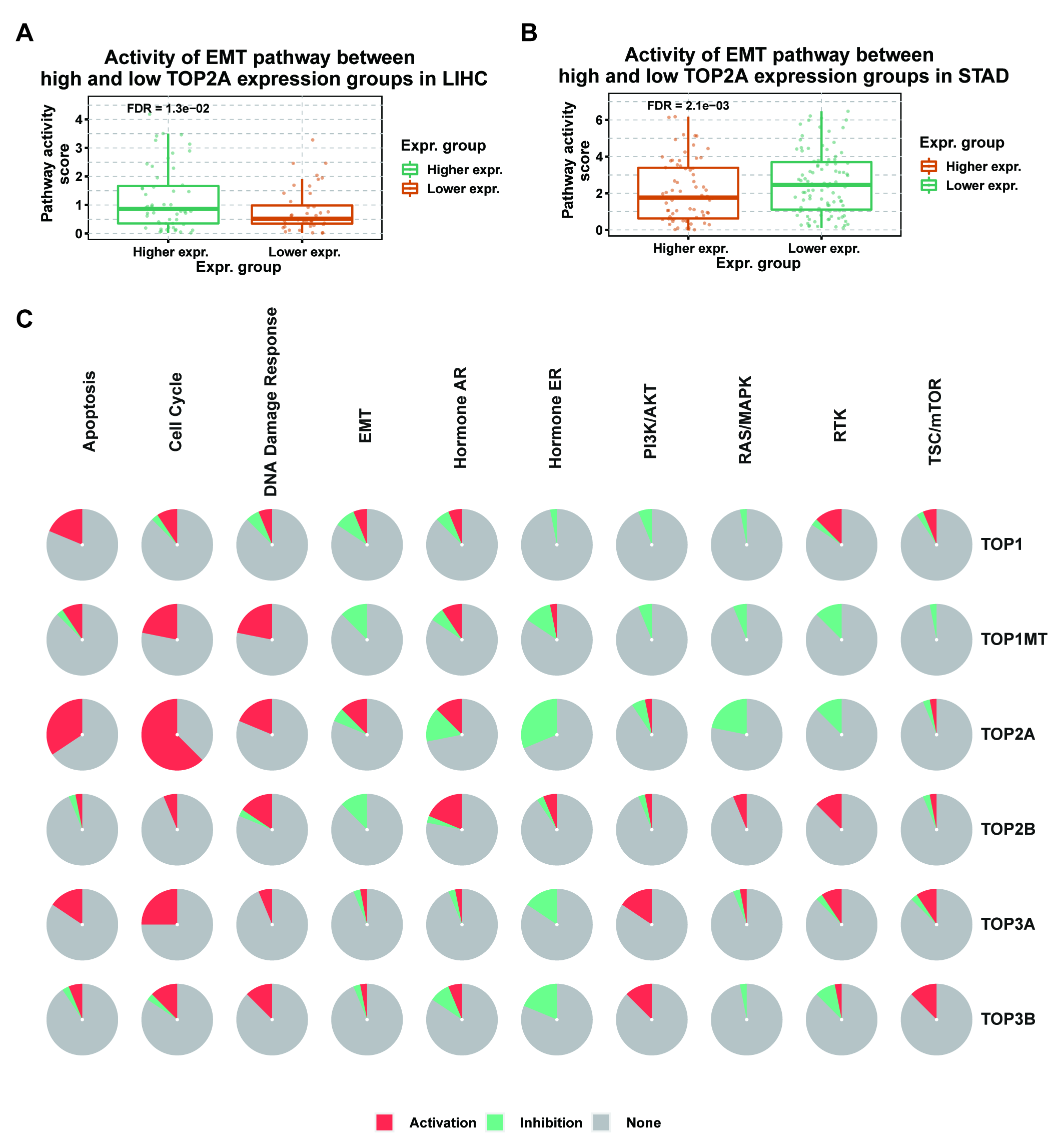

Supplement: S4 Fig — (A) The box chart showed the difference of EMT pathway activity score between TOP2A high expression group and TOP2A low expression group in LIHC. (B) The box chart showed the difference of EMT pathway activity score between TOP2A high expression group and TOP2A low expression group in STAD. (C) Pathway pie plot showing the global percentage of cancer types in which the specific topoisomerase family genes has an effect on the specific pathway in pan-cancer. (TIF) [file pone.0274546.s004.tif]

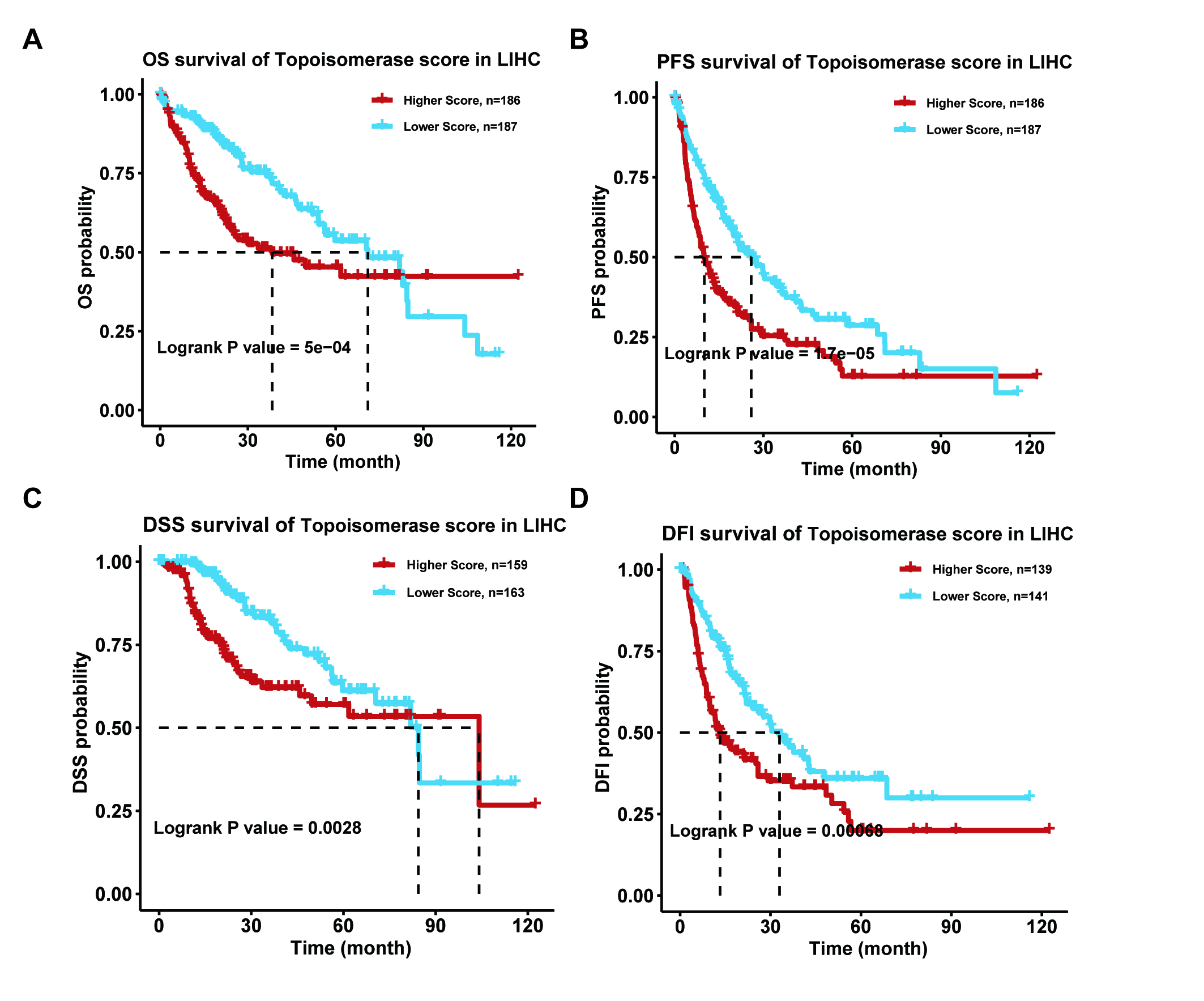

Supplement: S5 Fig — Kaplan-Meier curve showing the survival difference between high and low topoisomerase score in LIHC, including OS (A), PFS (B), DSS (C) and DFI (D). (TIF) [file pone.0274546.s005.tif]
